# Use of PB-Cre4 Mice for Mosaic Gene Deletion

**DOI:** 10.1371/journal.pone.0053501

**Published:** 2013-01-07

**Authors:** Andreas Birbach

**Affiliations:** Department of Vascular Biology, Center for Physiology and Pharmacology, Medical University of Vienna, Vienna, Austria; University of Nevada School of Medicine, United States of America

## Abstract

Transgene expression from short promoters in transgenic animals can lead to unwanted transgene expression patterns, often as a byproduct of random integration of the expression cassette into the host genome. Here I demonstrate that the often used PB-Cre4 line (also referred to as “Probasin-Cre”), although expressing exclusively in the male prostate epithelium when transmitted through male mice, can lead to recombination of loxP-flanked alleles in a large variety of tissues when transmitted through female mice. This aberrant Cre activity due to Cre expression in the oocytes leads to different outcomes for maternally or paternally transmitted loxP-flanked alleles: Maternally inherited loxP-flanked alleles undergo recombination very efficiently, making female PB-Cre4 mice an efficient monoallelic “Cre deleter line”. However, paternally inherited loxP-flanked alleles are inefficiently recombined by maternal PB-Cre4, giving rise to mosaic expression patterns in the offspring. This mosaic recombination is difficult to detect with standard genotyping approaches of many mouse lines and should therefore caution researchers using PB-Cre4 to use additional approaches to exclude the presence of recombined alleles. However, mosaic recombination should also be useful in transgenic “knockout” approaches for mosaic gene deletion experiments.

## Introduction

Transgene expression in transgenic animals is often achieved by a short promoter element which ideally should constrict gene expression to a specific tissue of interest. However, use of these small expression units and the classic transgenic technique of pronuclear injection lead to random integration of the unit in the genome. Consequently, transgene expression is not under sole control of the short promoter element, but also of the host locus. This leads to aberrant transgene expression patterns in many transgenic lines with expression in tissues not primarily targeted [1]. Use of larger constructs such as bacterial artificial chromosomes can help in controlling these effects [2]. For short transgenic constructs and random integration, it is often difficult to distinguish contributions of the promoter itself, the locus or a combinatorial effect of the promoter in its immediate surroundings to a specific transgene expression pattern in a single line. Use of multiple transgenic lines and/or cloning and analysis of the integration site can help in explaining the pattern.

The PB-Cre4 line has been created to achieve tissue specific gene deletions in the prostate epithelium [3]. This line uses the well-described ARR2PB promoter, comprising a proximal element of the rat *Probasin* (PB) promoter and two androgen responsive regions (ARR), to direct transgene expression of the Cre recombinase to prostate epithelial cells [4]. PB-Cre4 has been used in multiple studies to achieve gene recombination/deletion in the prostate epithelium [5], [6], [7], [8], [9], [10], [11], [12], [13]. The line has been extensively characterized and shows Cre recombinase activity in the prostate epithelium of all murine prostate lobes and rare, scattered recombination in the fibromuscular stroma. Additionally, a limited activity in a punctate pattern was seen in cells of the testis and the ovary. In this paper, I demonstrate that the activity in the oocyte follicles can be used to either completely ablate a loxP-flanked (“floxed”) allele, i.e. use PB-Cre4 as a deleter line, or to excise the floxed elements in a mosaic pattern, dependent on the maternal or paternal inheritance of the floxed allele.

## Materials and Methods

### Ethics Statement

Animal housing and sacrificing was in accordance with guidelines of the Medical University of Vienna and the Austrian Ministry of Science. The study was approved by the ethics committee of the Medical University of Vienna.

### Animals

PB-Cre4 mice [3] (official strain nomenclature: Tg(Pbsn-cre)4Prb) were obtained from the NCI Frederick mouse repository and had been backcrossed for more than 10 generations into a C57BL/6JHim (derived from the derived from the cohort of the Medical University of Vienna in Himberg, Austria) background.

Mice with loxP-flanked alleles of the tumor suppressor *Pten* were a gift of Prof. Tak Mak [14]. Mice with the reporter cassette (CAG-loxP-CAT-loxP-EGFP mice) were a gift from Prof. Jun-ichi Miyazaki [15]. All animals were kept on a C57BL/6JHim background.

### Genotyping PCRs

Genotyping was done on DNA isolated from tail biopsies. Tail biopsies (ca. 2 mm in length) were digested in 500 µl lysis buffer (400 mM NaCl, 0.1%SDS, 2 mM EDTA, 0.12 mg/ml proteinase K) for 3–24 hrs at 58°C. 200 µl 5 M NaCl was added, tail debris was pelleted (5′ 14,000 g), followed by DNA precipitation from the supernatant by addition of 1 volume isopropanol. DNA pellet was precipitated (30′ 14,000 g), washed with 70% Ethanol, dried and dissolved in 100 µl 10 mM Tris pH8. 1 µl of this solution was used for PCR.

**Figure 1:**
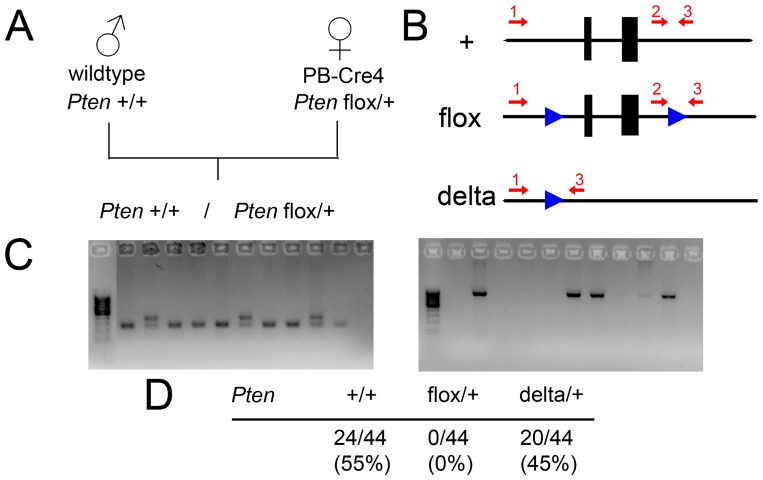
Maternal PB-Cre4 leads to efficient recombination of maternally transmitted loxP-flanked alleles. A, breeding scheme: male wildtype mice with two *Pten* wildtype alleles (marked “+”) were bred to female mice carrying the ARR2PB-Cre transgene (PB-Cre4 line) and a loxP-flanked *Pten* allele (“flox”) as well as a wildtype allele. The expected outcome (depicted here) concerning the *Pten* status of the offspring would be equal numbers of *Pten* +/+ (one wildtype allele each from the father and the mother animal) and *Pten* flox/+ (one wildtype allele from the father, one floxed allele from the mother) animals. B, Scheme of the *Pten* allele and primers (red arrows) used for their detection. A PCR using primers 2 and 3 yields a band for the+allele and a larger band for the floxed allele. In case of recombination, the sequence between the two loxP sites (blue triangles) is deleted, yielding a delta allele which can be detected by a PCR of primers 1 and 3. C, examples of PCRs with primers 2/3 (left) distinguishing between +/+ and flox/+ genotypes, and PCRs with primers 1/3, detecting delta alleles (right). For the left image, tail biopsies from offspring mice where one parental animal carried a *Pten* flox allele and none carried the PB-Cre4 transgene were used, while for the right image, tail biopsies from offspring mice of the breeding indicated in A were used. D, In the offspring of the breeding in A, no flox/+ combination was detected, but delta/+ was seen in nearly equal numbers as +/+.

Primers for *Pten* genotyping were (according to the scheme in Figure 1): Primer 1: 5′-GGT AGA CTA GTC GGT ACT CAG-3′, Primer 2: 5′-CTCCTCTACTCCATTCTTCCC-3′, Primer 3: 5′-ACTCCCACCAATGAACAAAC-3′. PCR was done for 35 cycles, with annealing temperature of 55°C, and elongation time 60″ at 72°C.

### Cryosectioning and Microscopy

Mice were sacrificed at 4–8 weeks of age, tissues removed and fixed in 4% paraformaldehyde in 1 x phosphate buffered saline (PBS) at pH = 7–7.4. After fixation for 1–2 hours, tissues were transferred to 20% sucrose/1xPBS and incubated overnight at 4°C. Tissues were then transferred to TissueTek (Sakura Finetek) and shock-frozen in liquid N2, then stored at −80°C until cutting. Eight-micrometer sections were cut using a Microm HM500 OM Cryostat and mounted onto SuperFrost Plus slides (Thermo Scientific). Slides were dried for 2 hours at room temperature, washed several times in PBS, and Hoechst 33342 (bisbenzimide; Sigma) was included in the last wash at 100 ng/ml to counterstain nuclei. Samples were embedded in PBS/glycerol (1∶7), overlaid with coverslips, and sealed with nailpolish. Images were acquired on an Olympus AX470 microscope equipped with an F-View II (grayscale) digital camera using the manufacturer’s software (CellP) and appropriate filter sets for fluorescence.

### Genome Walking

For cloning upstream sequences of the transgenic cassette, 2.5 µg genomic DNA was digested with one of a number of blunt-end restriction enzymes (AluI, DraI, SmaI, EcoRV, PvuII) in a total volume of 100 µl. DNA was purified by Phenol/Chloroform extraction followed by ethanol precipitation. Adaptors were as follows: 5′-GTAATACGACTCACTATAGGGCACGCGTGGTCGACGGCCCGGGCTGGT -3′ and 5′- (PO4)ACCAGCCC(NH2)-3′, whereby PO4 means phosphorylation at the 5′ terminus and NH2 amination at the 3′ terminus. Adaptors were annealed by heating a 1∶1 mixture of the adaptor oligonucleotides to 100°C and allowing to slowly cool to room temperature. Adaptors were ligated to the digested genomic DNA using concentrated T4 DNA ligase (New England Biolabs).

The first PCR was done with primers 5′-GTAATACGACTCACTATAGGGC-3′ (within the adaptor sequence) and 5′- CTAGGCATGGACAATGCCCAATGC-3′ (within ARR2PB) and the following cycle conditions: 94°C 2′, 7 cycles of: 94°C 25″ –72°C 3′, 32 cycles of: 94°C 25″–67° 3′, 67°C 7′. 1 µl of the diluted (1∶50) PCR was used as template for the nested PCR, using primers 5′-ACTATAGGGCACGCGTG-3′ (within the adaptor) and 5′-CCCAATGCCTGTCCCATTCTTCAGGCAT-3′ (within ARR2PB) and the following cycle conditions: 94°C 2′, 5 cycles of 94°C 25″ –72°C 3′, 30 cycles of 94°C 25″ –67°C 3′, 67°C 7′.

Integration site was confirmed with primers 5′-GAT GAA GCC CAG GTG CCA GTT G-3′ (Chromosome 11) and 5′-CCC AAT GCC TGT CCC ATT CTT CAG GCA T-3′ (ARR2PB). Annealing temperature for this PCR was 64°C, the PCR was run for 35 cycles with an elongation time (at 72°C) of 90″.

## Results

### Maternal PB-Cre4 Leads to Full Deletion of Maternally Transmitted Loxp-flanked Alleles

My group has used PB-Cre4 mice to establish a model of inflammatory tumor development in the prostate [16]. While breeding the animals for this model, we observed unexpected phenotypes in a subset of mice, including tumor developments in other organs than the prostate. I suspected aberrant Cre activity to be the cause for these phenotypes. PB-Cre4 has been shown to be active in a small number of cells in the testis (seminiferous tubules) as well as the ovary (oocytes) [3]. In order to verify this hypothesis, I set up a number of test breedings using PB-Cre4 line together with a line carrying a loxP-flanked allele of the tumor suppressor *Pten* used in our studies. For analysis of the genotype, a PCR distinguishing the normal, i.e. “wildtype” allele from the loxP-flanked (“floxed”) allele was used as well as a PCR detecting an excised (“delta”) allele of *Pten* (Figure 1). When breeding male mice carrying PB-Cre4 in addition to a *Pten* flox allele and a *Pten* wildtype allele to wildtype females, a normal distribution of the *Pten* flox and *Pten* wildtype allele in the offspring was observed. No delta allele was detected (not shown). However, when breeding wildtype male mice to female mice carrying the PB-Cre4 transgene as well as *Pten* flox and wildtype alleles, the floxed allele was never transmitted. Instead, a fraction of the offspring carried a delta allele in the tail biopsies (Figure 1). This indicates that Cre activity in the oocytes is sufficient to induce recombination. Thus, female PB-Cre4 mice can be used for efficient deletion of maternally inherited loxP-flanked DNA elements. In our hands, the efficiency is likely close to 100% (the floxed allele was never detected), while the earlier characterization of PB-Cre4 mentioned transmission of the floxed allele in about half of the cases.

### Maternal PB-Cre4 Mediates Mosaic Deletion of Paternally Transmitted LoxP-flanked Alleles

Expression of PB-Cre4 in the oocyte leads to efficient deletion of a loxP-flanked allele present in the oocyte genome. When breeding female PB-Cre4 mice with oocyte Cre activity to male mice carrying a loxP-flanked allele, the situation is less predictable and there are three possible outcomes: One, Cre levels in the fertilized oocyte are insufficient to mediate recombination, and the loxP-flanked allele is transmitted as such, with no delta allele detectable. Two, Cre level/activity in the fertilized oocyte is sufficient to mediate recombination, and loxP-flanked alleles are always transformed into delta alleles. Three, Cre is present in the fertilized oocyte, but levels are in a range that they do not always lead to recombination of the floxed allele. Instead, the stochastic process of Cre-mediated deletion takes place in single cell(s) of the multicellular early embryo. In the latter case, this would lead to a mosaic animal consisting of cells with both unrecombined (floxed) and recombined (deleted) alleles.

**Figure 2:**
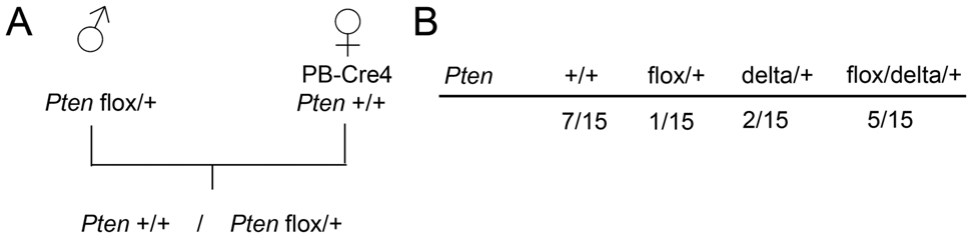
Maternal PB-Cre4 leads to mosaic deletion of paternally transmitted loxP-flanked alleles. A, breeding scheme. Male mice carrying a *Pten* flox and a *Pten*+allele were bred to female mice carrying the Cre transgene. In the absence of aberrant Cre activity, an equal distribution of *Pten* +/+ and *Pten* flox/+ offspring is expected. B, results from the breeding: Maternal PB-Cre4 and paternal *Pten* flox/+ lead to offspring in which the floxed allele is still present in roughly the expected frequency in tail biopsies, but a delta allele can be detected as well, indicating a mosaic deletion.

In order to distinguish between these possibilities, male mice carrying a *Pten* flox and a *Pten* wildtype allele were bred to female mice carrying PB-Cre4. I obtained mice with the wildtype allele, mice carrying the floxed allele or delta allele and mice carrying both the floxed and the delta allele according to the PCRs from tail biopsies (Figure 2). This indicates that the third possibility is valid and Cre activity is limited to produce mosaic animals.

### Mosaicism can be Visualized in a Variety of Tissues

**Figure 3:**
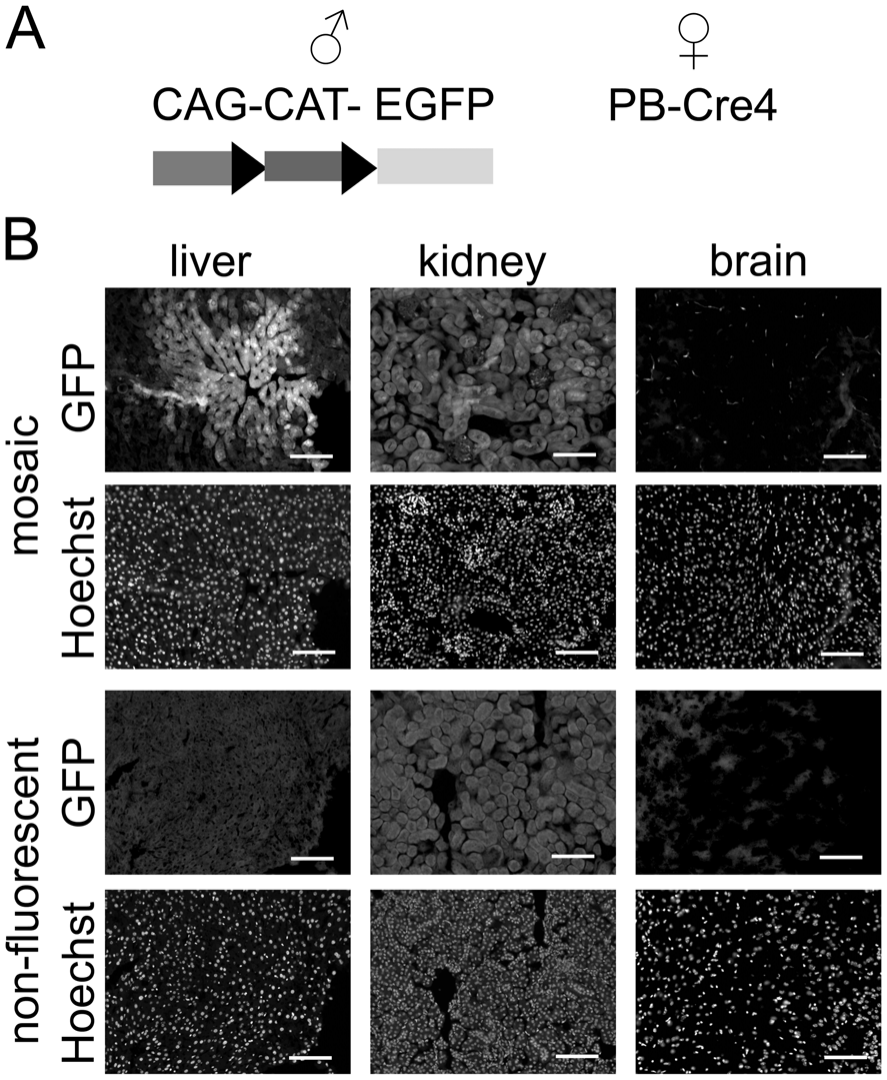
Maternal PB-Cre4 leads to mosaicism of paternally transmitted alleles which can be visualized in reporter animals (part I). A, female mice carrying the Cre transgene were bred to male mice carrying one allele of CAG-loxP-CAT-loxP-EGFP, a construct expressing the CAT gene from a constitutive promoter (CAG). The CAT gene is loxP-flanked (triangles), and upon Cre-mediated recombination, this allele will express EGFP. B, different organs (here: liver, kidney, brain cortex) show EGFP fluorescence in a mosaic pattern in an offspring animal from this breeding. Tissue sections from an animal not expressing EGFP (“non-fluorescent”) are shown as controls to highlight tissue autofluorescence. Hoechst staining is shown to demonstrate tissue architecture. Scale bars, 100 µm.

**Figure 4:**
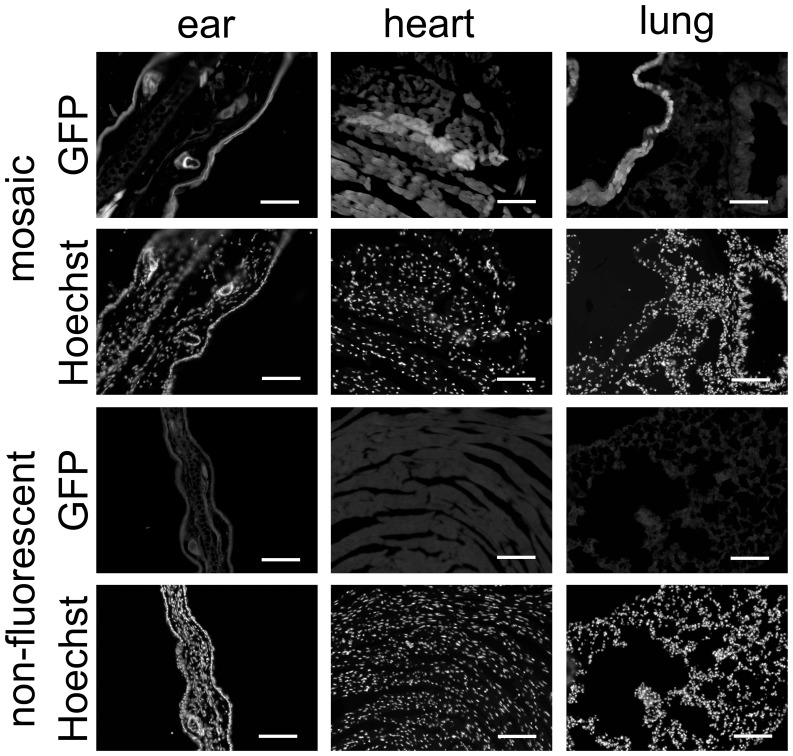
Maternal PB-Cre4 leads to mosaicism of paternally transmitted alleles which can be visualized in reporter animals (part II). Organs from the same animal as in Figure 3 are shown to demonstrate mosaicism in a variety of tissues. Controls were used as in Figure 3. Scale bars, 100 µm.

In order to demonstrate mosaicism in a range of tissues and resolve it on a cellular level than just in a DNA sample of multiple cells, the breeding approach was changed. Female PB-Cre4 mice were bred to male mice carrying an allele with a loxP-flanked Stop cassette followed by a cDNA coding for EGFP. This expression unit is placed behind a ubiquitous promoter, meaning that EGFP is expressed in a variety of tissues following Cre-mediated deletion of the Stop cassette. Offspring from these breedings were sacrificed at young adulthood and cryosections prepared as described in Methods. Mosaicism was seen in all organs examined (Figure 3, Figure 4; Figure S1, Figure S2). However, detection was difficult in some cell types due to lower expression levels of the EGFP transgene. Reporter gene expression was seen in cells of different developmental origins such as epithelial kidney or skin cells and smooth or cardiac muscle cells. If mosaicism were to occur by recombination of a single cell at a multi-cell stage, one would expect the daughter cells arising from the recombined cells to form groups in the adult tissue. This pattern was seen in organs like the liver, the kidney or the lung, while expression patterns in the brain appeared to be more scattered. This could indicate cell migration during brain development.

**Figure 5:**
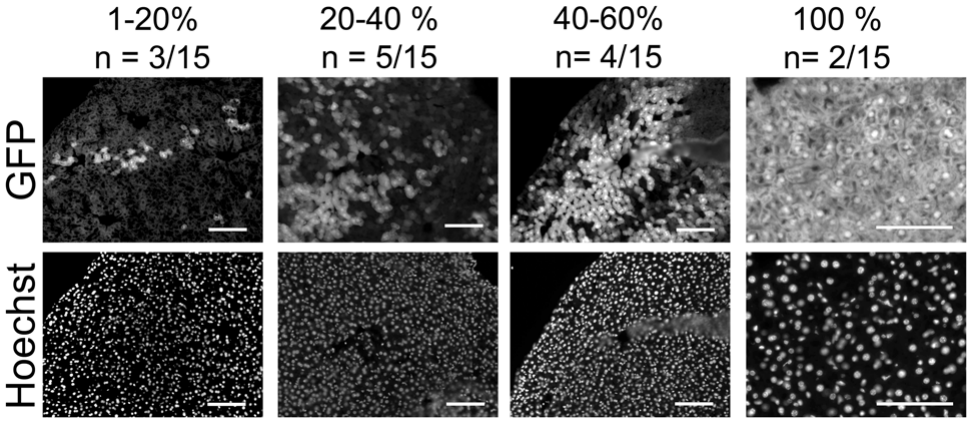
Different degrees of mosaicism in transgenic livers. The numbers above the images indicate the percentage of EGFP positive cells (top row) and the numbers of animals (out of n = 15 animals with any degree of mosaicisim, i.e. EGFP fluorescence) within the respective group (row below). Examples for tissues of the respective groups are shown below the numbers. Scale bars, 100 µm.

The liver was the organ with the best signal to noise ratio. Therefore I decided to quantify the degree of mosaicism with sections of this organ from different mice. Slightly more than half of the offspring animals did not show EGFP fluorescence. This is expected because the male animals in breedings carried only one allele of the CAG-loxP-CAT-loxP-EGFP transgene. Fluorescent liver tissues (n = 15) showed different degrees of mosaicism. A fraction showed EGFP fluorescence only in a few cells within a tissue section, while I also found livers with apparent full recombination. A majority of tissues showed an intermediate degree of mosaicism (Figure 5, Figure S3).

### The ARR2PB-Cre Transgene Cassette is Integrated Close to the Telomeric End of Chromosome 11q

In order to determine the reasons for aberrant Cre activity, i.e. evaluate whether Cre expression in the oocyte was due to the transgene integration site or the promoter, I attempted to determine the PB-Cre4 integration locus.

**Figure 6:**
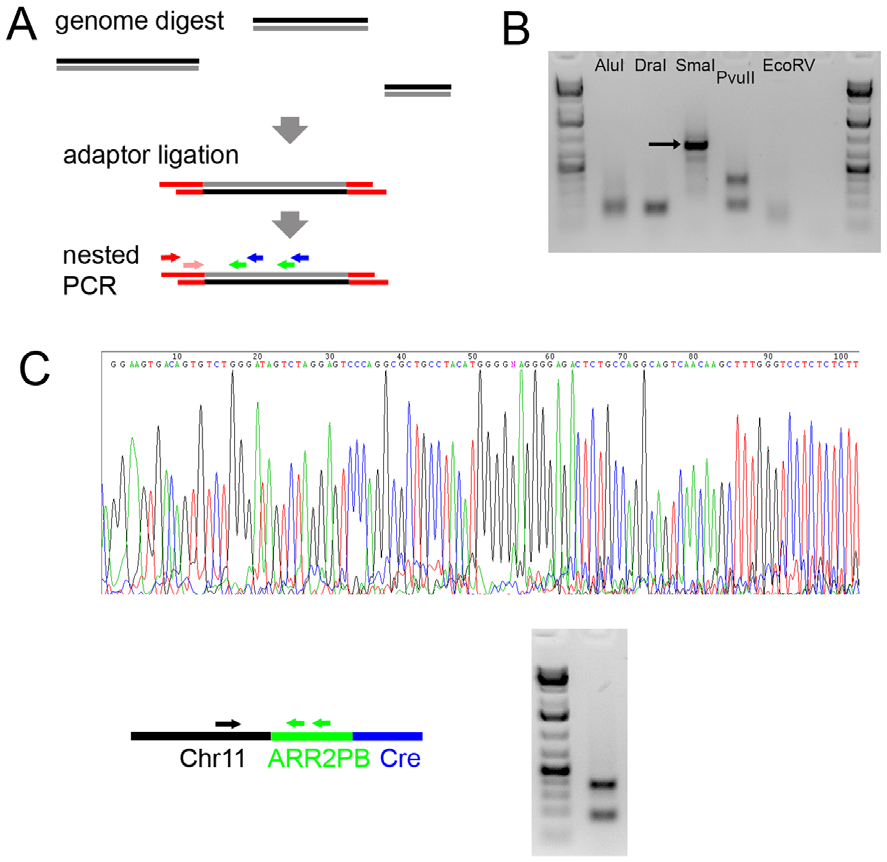
Cloning of the PB-Cre4 integration site. A, scheme of the genome walking procedure used. Genomic DNA was cut using blunt-end cutters, followed by ligation of adaptor oligonucleotides. Nested PCR was done using primers within the adaptors as well as gene-specific primers. The gene-specific primers in the ARR2PB sequence primed twice because of the two identical androgen-responsive regions. The corresponding results for different blunt-end cutters are shown in B. Bands obtained for SmaI and PvuII were sequenced. The sequence for the SmaI band (arrow) is shown in C. D, PCR primers were designed within the putative flanking (upstream) sequence and within the ARR2PB (again priming twice), yielding a double band as expected. Bands were sequenced again, confirming the integration site.

The upstream sequence of the integrated ARR2PB-Cre construct was cloned using a modified “genome walking” procedure [17]. The method is briefly explained in Figure 6 (for details, see Materials and Methods). It is based on digestion of genomic DNA with various restriction enzymes creating blunt ends and ligation of adaptors onto the differently sized fragments, followed by PCR and nested PCR using primers within the adaptors and the starting sequence (in this case, the ARR2PB sequence). The resulting PCR fragments were sequenced. I identified a genomic sequence on murine chromosome 11 (GRCm38:11:120521381 (ENSEMBL release 69) according to ENSEMBL nomenclature) next to the transgenic ARR2PB sequence. This locus at the long arm of chromosome 11 (11q) is close to the telomeric end. In order to verify this putative integration site, PCRs with primers unrelated to the cloning procedure were run to confirm a link between the chromosome 11 locus and the ARR2PB-Cre sequence (Figure 6). It is very unlikely that a second, independent integration site is present in this line, because the mice have been bred for more than 10 generations in our lab and the integration site according to PCR is present in all animals tested, including the female PB-Cre4 mice which showed the phenomenon of complete or partial recombination in the offspring.

The identified locus has not been described as being expressed in the oocyte or early embryo. I checked the expression of the genes closest to the integration site, *Slc25a10* (a mitochondrial carrier) and *Gcgr* (glucagon receptor) in silico. The NCBI UniGene EST (expressed sequence tag) database (http://www.ncbi.nlm.nih.gov/unigene) did not show any ESTs for these genes in the fertilized ovum or ovary. No expression in the ovary was also found in the BioGPS database (http://www.biogps.org). Thus aberrant Cre activity in the oocyte may be unrelated to the integration locus.

## Discussion

This report has implications on different levels. First, it demonstrates the usefulness as well as the limitations of using PB-Cre4 for prostate-specific gene deletions, urging against using female mice carrying the transgene for breedings. Particularly, the finding of mosaicism of paternally inherited floxed alleles is important in this regard. The following example highlights the reason for this warning: Most transgenic breedings are controlled by a PCR approach distinguishing between a “wildtype” and a “floxed” allele. While complete deletion as in the case of maternally inherited loxP-flanked alleles will be detected after some time due to the absence of PCR bands for these alleles, the mosaic deletion will not. In case of mosaicism, both wildtype and floxed alleles will be present in the offspring in roughly correct ratios, while the deleted (“delta”) allele is usually not checked for in genotypings. Thus, offspring is then attributed either a wildtype or floxed genotype of a specific allele, while in addition carrying a mosaic deletion which may cause a phenotype. Theoretically, this could lead to attribution of a specific prostate phenotype to epithelial gene deletion, while the phenotype only arises due to (additional) mosaic deletion in the fibromuscular stroma or infiltrating immune cells. Therefore it is recommended that researchers having used PB-Cre4 in their work and not paid attention to transmit the Cre gene only from the male animals in breedings re-evaluate their findings in this respect. It has to be noted, however, that PB-Cre4 when only transmitted by males is a safe and accurate way to achieve prostate-specific gene deletion in my hands as well as in multiple literature reports. In case that transmission from both male and female breeding partners is wanted, e.g. when doing complex multiple transgenic animals, it is recommended to use one of the inducible prostate-specific Cre lines [18], [19]. These lines have been shown to be epithelium-specific and therefore offer an additional advantage compared to PB-Cre4. The PSA-Cre line is another strain valuable in this regard [20].

Second, the finding that maternal PB-Cre4 leads to mosaic deletion of paternally transmitted floxed alleles, while completely deleting maternally transmitted floxed alleles, is useful for transgenic research. Studying mosaic deletion patterns by the use of reporter genes, as initiated in this study, can be interesting for developmental biology in studying contributions of cells of the early embryo to the adult tissues as well as in studying cell migration during development. Mosaic deletion can also be useful for classical gene deletion (“knockout”) experiments. For example, if a researcher wants to study a gene of interest (*Gene A*) in multiple adult tissues and complete gene deletion (*Gene A* −/−) is embryonically lethal, the researcher may want to consider mosaic deletion of a floxed allele on top of a heterozygous (*Gene A* +/−) background. This could circumvent embryonic lethality and allow studying gene function in multiple tissues without the need for multiple tissue-specific Cre lines.

Third, identification of the integration site of PB-Cre4 has implications for users of this line. While the integration site is not in a locus known for expression in the oocyte or early embryo, it cannot be ruled out that integration of the promoter in the microenvironment creates binding sites for transcriptional activators active in the oocyte. Moreover, it is still possible that the ARR2PB promoter itself leads to expression in the ovarian follicles, although ARR2PB-CAT transgenic mice did not show reporter gene activity in the ovary [4]. The same paper demonstrates that the ARR2PB promoter is not only activated by the androgen receptor, but also by other nuclear receptors like the glucocorticoid receptor. It is possible that nuclear receptor activity in the oocyte leads to expression from the promoter. Limited expression levels in a small number of cells, such as oocytes within the ovary, could be difficult to detect but still sufficient to induce Cre-mediated recombination. The integration site at Chromosome 11 (GRCm38:11:120,521,381) also has implications for bi-allelic gene deletion with PB-Cre4: due to genetic linkage, genes with loci close to the integration site will show a decreased breeding efficiency (i.e., probability of a suitable crossing over event) in compound mice with loxP-flanked alleles for these genes and PB-Cre4. For example, the site is about 20 megabases off the loci for *Stat3* and *Stat5*, two well-known signaling genes in cancer. The locus for *Socs3*, also implicated in prostate cancer, is just 2 megabases away from the integration site. Considering that 1 centimorgan roughly corresponds to 1 megabase in the mouse genome, the efficiency for a chromosome carrying both PB-Cre4 and *Socs3* flox in the offspring would be just 2%.

In conclusion, the implications of the presented findings can be summarized in three short statements:

1. PB-Cre4 will faithfully maintain prostate-specific gene deletion only when transmitted through male animals; transmission through female animals causes complete or mosaic deletion which can be easily overlooked using common genotyping strategies.
2. The mosaic gene deletion of paternally transmitted loxP-flanked alleles by maternal PB-Cre4 can be a useful tool for gene deletion experiments and developmental biology.
3. The integration site of the transgene should be taken into consideration when targeting alleles close to the integration site.

## Supporting Information

Figure S1 **Maternal PB-Cre4 leads to mosaicism of paternally transmitted conditioned alleles (part I).** Colored overlays of images in Figure 3 are shown.
(TIF)

Figure S2 **Maternal PB-Cre4 leads to mosaicism of paternally transmitted conditioned alleles (part II).** Colored overlays of images in Figure 4 are shown.
(TIF)

Figure S3 **Different degrees of mosaicism in transgenic livers.** Colored overlays of images in Figure 5 are shown.
(TIF)
