# Supplementary figures and images for: Use of PB-Cre4 Mice for Mosaic Gene Deletion
Source: PLoS One. 2013 Jan 7;8(1):e53501. doi: 10.1371/journal.pone.0053501 (PMC3538545; doi:10.1371/journal.pone.0053501)

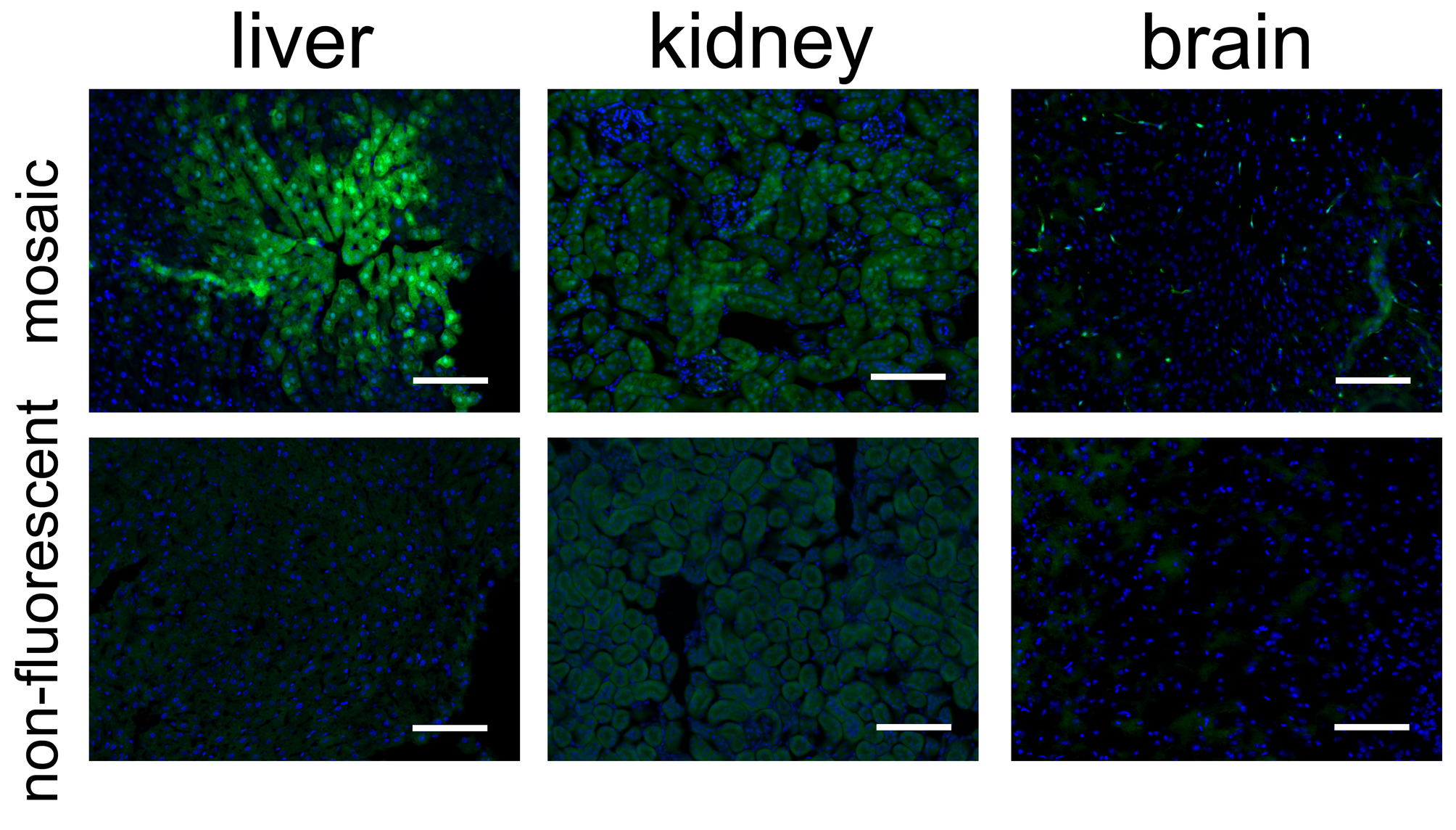

Supplement: Figure S1 — Maternal PB-Cre4 leads to mosaicism of paternally transmitted conditioned alleles (part I). Colored overlays of images in Figure 3 are shown. (TIF) [file pone.0053501.s001.tif]

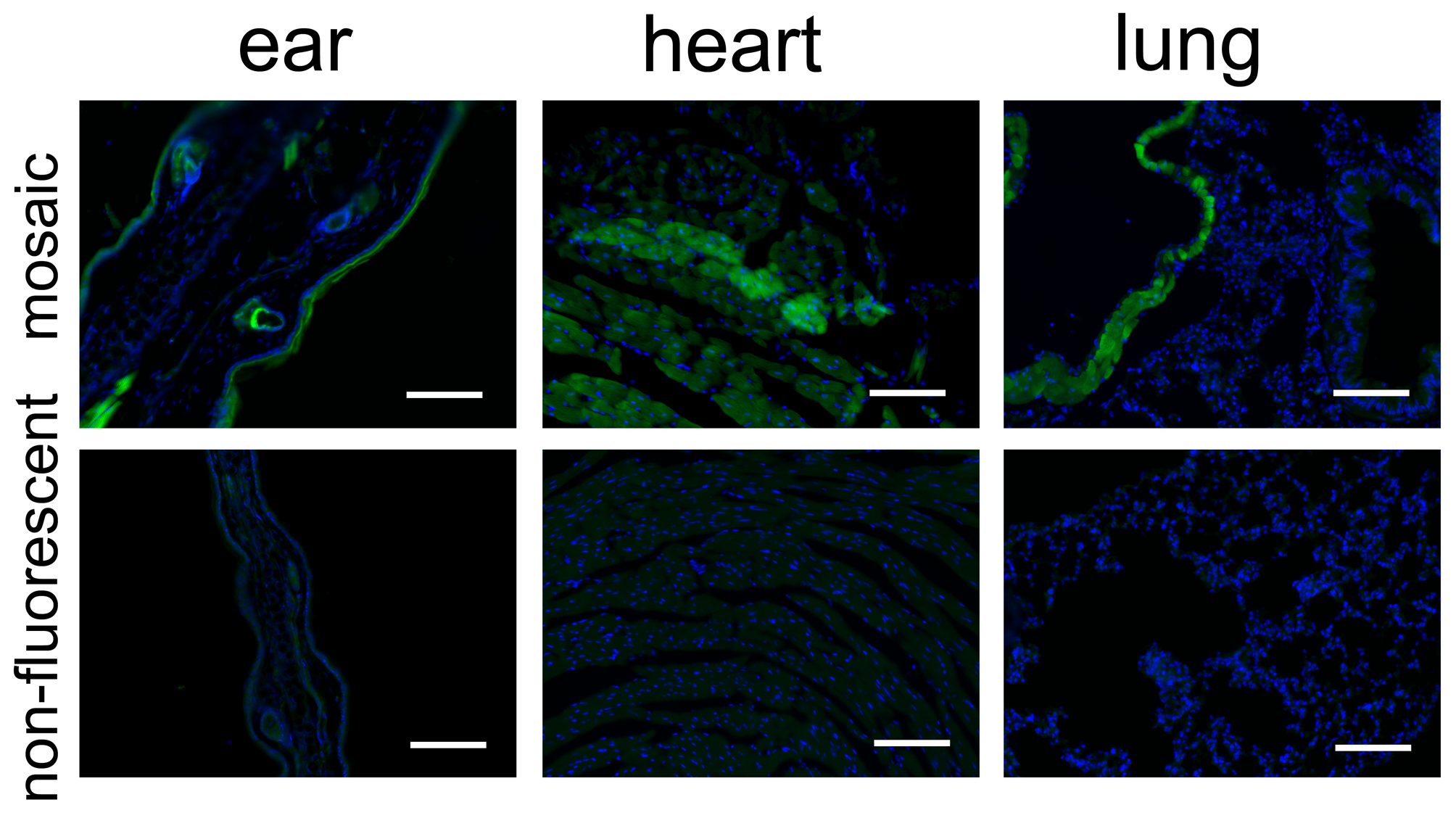

Supplement: Figure S2 — Maternal PB-Cre4 leads to mosaicism of paternally transmitted conditioned alleles (part II). Colored overlays of images in Figure 4 are shown. (TIF) [file pone.0053501.s002.tif]

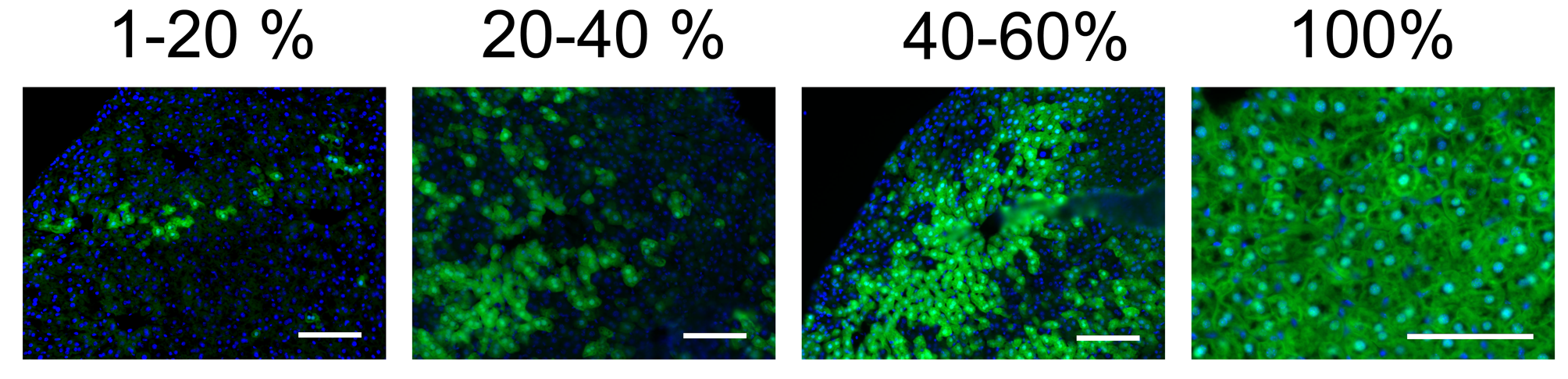

Supplement: Figure S3 — Different degrees of mosaicism in transgenic livers. Colored overlays of images in Figure 5 are shown. (TIF) [file pone.0053501.s003.tif]
